# Genome-Wide Association Mapping and Genomic Prediction of Anther Extrusion in CIMMYT Hybrid Wheat Breeding Program via Modeling Pedigree, Genomic Relationship, and Interaction With the Environment

**DOI:** 10.3389/fgene.2020.586687

**Published:** 2020-12-08

**Authors:** Anil Adhikari, Bhoja Raj Basnet, Jose Crossa, Susanne Dreisigacker, Fatima Camarillo, Pradeep Kumar Bhati, Diego Jarquin, Yann Manes, Amir M. H. Ibrahim

**Affiliations:** ^1^Texas A&M University, College Station, TX, United States; ^2^Department of Horticulture, University of Wisconsin, Madison, WI, United States; ^3^International Maize and Wheat Improvement Center (CIMMYT), Texcoco, Mexico; ^4^Borlaug Institute for South Asia (BISA), Ludhiana, India; ^5^Department of Agronomy and Horticulture, University of Nebraska, Lincoln, NE, United States; ^6^Syngenta Seeds, Saint-Sauveur, France

**Keywords:** GWAS, anther extrusion, floral traits, hybrid wheat, genome-wide prediction

## Abstract

Anther extrusion (AE) is the most important male floral trait for hybrid wheat seed production. AE is a complex quantitative trait that is difficult to phenotype reliably in field experiments not only due to high genotype-by-environment effects but also due to the short expression window in the field condition. In this study, we conducted a genome-wide association scan (GWAS) and explored the possibility of applying genomic prediction (GP) for AE in the CIMMYT hybrid wheat breeding program. An elite set of male lines (*n* = 603) were phenotype for anther count (AC) and anther visual score (VS) across three field experiments in 2017–2019 and genotyped with the 20K Infinitum is elect SNP array. GWAS produced five marker trait associations with small effects. For GP, the main effects of lines (L), environment (E), genomic (G) and pedigree relationships (A), and their interaction effects with environments were used to develop seven statistical models of incremental complexity. The base model used only L and E, whereas the most complex model included L, E, G, A, and G × E and A × E. These models were evaluated in three cross-validation scenarios (CV0, CV1, and CV2). In cross-validation CV0, data from two environments were used to predict an untested environment; in random cross-validation CV1, the test set was never evaluated in any environment; and in CV2, the genotypes in the test set were evaluated in only a subset of environments. The prediction accuracies ranged from −0.03 to 0.74 for AC and −0.01 to 0.54 for VS across different models and CV schemes. For both traits, the highest prediction accuracies with low variance were observed in CV2, and inclusion of the interaction effects increased prediction accuracy for AC only. In CV0, the prediction accuracy was 0.73 and 0.45 for AC and VS, respectively, indicating the high reliability of across environment prediction. Genomic prediction appears to be a very reliable tool for AE in hybrid wheat breeding. Moreover, high prediction accuracy in CV0 demonstrates the possibility of implementing genomic selection across breeding cycles in related germplasm, aiding the rapid breeding cycle.

## Introduction

Hybrid wheat offers great promise in terms of higher grain yield and stability across a wide range of wheat-producing environments globally (Gowda et al., 2010, 2012; Mühleisen et al., 2014; Zhao et al., 2015; Basnet et al., 2019; Adhikari et al., 2020a; Easterly et al., 2020). Hybrid wheat has gotten attention in private and public sector breeding programs since the 1950s, but it has not led to any considerable commercial success (Virmani and Edwards, 1983; Longin et al., 2012; Adhikari et al., 2020a). There has been a growing interest in hybrid wheat research again in the last decade in North America (Dreisigacker et al., 2005; Basnet et al., 2019; Adhikari et al., 2020a,b; Easterly et al., 2020) and Europe (Gowda et al., 2012; Longin et al., 2012; Zhao et al., 2015). However, hybrid wheat varieties are currently commercially marketed only in Europe and some parts of India and China, and acreages are fairly low in all three areas (Longin et al., 2012; Gupta et al., 2019).

The most vital limitation for the commercial success of hybrid wheat has always been the complexity and cost of hybrid seed production even though chemical-based sterility, cytoplasmic male sterility, and genetic male sterility systems with varying levels of efficiency are available (Virmani and Edwards, 1983; Longin et al., 2012). Irrespective of the method of hybrid seed production, one of the major factors that determines hybrid seed set is the outcrossing ability, which is determined by pollen load released by male parents upon flowering outside the floral structure (De Vries, 1971; Whitford et al., 2013) and opening of flowers in the female parents (Longin et al., 2014). Wheat flowers are cleistogamous, and extrusion of anthers outside the floral structure is a highly correlated proxy of pollen mass release that determines hybrid seed production (Whitford et al., 2013; Langer et al., 2014; Boeven et al., 2016, 2018). Hence, inheritance of anther extrusion (AE) in the context of hybrid seed production plays a key role and has, therefore, been extensively studied in Europe and North America (Langer et al., 2014; Boeven et al., 2016, 2018; Muqaddasi et al., 2017a,c,b, 2019).

AE was previously thought to be inherited in an oligogenic manner (Sage and De Isturiz, 1974). However, recent studies on the inheritance of AE with the use of high-density molecular markers have found the trait to be complex and quantitative (Boeven et al., 2016). Several groups have studied the inheritance of AE in the context of Fusarium head blight (caused by *Fusarium* species, including *Fusarium graminearum*, *F. culmorum*, *F. avenaceum*, *F. poae*, and *Microdochium nivale*) resistance and found similar results (Skinnes et al., 2010; Lu et al., 2013; Buerstmayr and Buerstmayr, 2015; Steiner et al., 2019). Genome-wide association studies (GWAS) and bi-parental mapping have identified dwarfing genes/alleles, also referred to as “reduced height loci” (*Rht*), present in modern wheat germplasm as the major loci or co-localized with major loci governing AE and pollen mass in wheat (Lu et al., 2013; Boeven et al., 2016; He et al., 2016). The *Rht* genes (*Rht-B1* and *Rh-tD1*) that cause plant height reduction also shorten the anther filaments and negatively impact AE (Boeven et al., 2016). Another height reduction loci *Rht24*, which does not reduce AE, has been suggested as an alternative to the *Rht1* loci for use in male lines in hybrid breeding (Würschum et al., 2018). Other than the *Rht* genes, previous studies reported the presence of several minor effect loci positively and negatively associated with AE in European winter wheat (Muqaddasi et al., 2017a,b) and CIMMYT spring wheat (Muqaddasi et al., 2019). Despite the identification of marker-trait associations and QTL, their application in hybrid wheat breeding via marker-assisted selection (MAS) for extending AE has limited scope since they are of very modest effects. Moreover, the *Rht* alleles have a very important role in height reduction of the wheat plant that is paramount for maintaining grain yield via prevention of lodging. *Rht* alleles have been widely deployed since the green revolution and are ubiquitous in elite CIMMYT germplasm (Ogihara et al., 2013; Aisawi et al., 2015; Basnet et al., 2019). These genes cannot be excluded for the sole purpose of increasing AE. In this context, genome-wide prediction or genomic selection (GS) appears to be the best strategy to breed for AE by exploiting the cumulative effect of many effect loci scattered throughout the genome (Meuwissen et al., 2001).

GS via whole-genome regression methods uses the information from thousands of molecular markers to capture not only major-effect genes but also the contribution of genomic regions with small effects (Meuwissen et al., 2001). GS has been utilized extensively in animal breeding and plant breeding to predict traits with complex genetic architecture using the information from molecular markers and pedigree information (Hayes et al., 2009; Crossa et al., 2017). In the case of multi-environment trials (METs) in plant breeding, GS models did not explicitly model G × E information since the phenotypic data from METs were analyzed separately to derive single phenotypic estimates, and they were used as single trait in a genomic estimated best linear unbiased predictor (G-BLUP) model (Burgueño et al., 2012). G-BLUP models have been extended for a multi-environment setting by Burgueño et al. (2012), where genetic correlations are used to explicitly model G × E. Jarquín et al. (2014) extended the G × E model to include environmental covariates both as main effects and interaction effects with genotypes and locations in a reaction norm model. The reaction norm model has been used extensively to predict complex traits with multi-environment data in wheat and cotton and was found to reveal higher prediction accuracies than single-trait G-BLUP and multi-trait G-BLUP models (Jarquín et al., 2014; Pérez-Rodríguez et al., 2015; Sukumaran et al., 2017, 2018). GS has been used to predict AE using single-trait G-BLUP (Boeven et al., 2016; Muqaddasi et al., 2017c). However, G × E and reaction norm models have not been tested thus far, despite AE showing high levels of G × E.

This study aims to (i) explore the wheat genome for major effect QTL associated with AE via GWAS and (ii) apply reaction norm G × E models to predict AE in a multi-environment setting with the goal of driving genetic gain for AE in the CIMMYT hybrid wheat breeding program.

## Materials and Methods

### Plant Materials and Field Experiments

The study consisted of 603 advanced parental lines from the CIMMYT hybrid wheat breeding program. The lines were planted in 2-m-long, double-row linear plots, with 20-cm inter-row spacing at El Batan, Mexico (20.83° N, 100.83° W) in 2017 and 2019, and at Obregon, Mexico (27.48° N, 109.93° W) in 2018 growing cycles. The trials within a location were unreplicated, and plants/spikes per plant were used as biological replicates (i.e., two to three individual plants per genotype and one to three spikes per plant). In each experiment, four or five random spikes from different plants in each plot were tagged prior to flowering, and two male floral traits, as they relate to AE, AE visual scores (VS), and extruded anther count (AC), were taken from the plots at flowering and post-flowering stages, respectively. Trapped anthers in two lateral florets (first and second florets) from six to eight middle spikelets were counted in each randomly tagged spike, and then the deduced AC was expressed as a percentage by using the formula by Boeven et al. (2016):

(1) $$\mathrm{Anther}\mathrm{extrusion}\left(\%\right)=\frac{\mathrm{Total}\,\mathrm{extruded}\,\mathrm{anthers}}{\mathrm{Total}\,\mathrm{number}\,\mathrm{of}\,\mathrm{anthers}}\times 100\%$$

For VS, a score of 1 to 10 was assigned to each genotype during flowering based on visual observations, where 1 indicates no anther extrusion and 10 indicates maximum anther extrusion. AC data were collected from all three environments, whereas VS was collected from only 2018 and 2019 experiments. In 2017, we missed the critical time during flowering to collect reliable VS data.

### Statistical Analysis of Phenotypic Data

Phenotypic data of AC from each trial were analyzed separately using software package META-R (Alvarado et al., 2015). Best linear unbiased estimates (BLUEs) were calculated for each genotype, considering the effects of genotypes as fixed and the effects of environments as random. In addition to the BLUEs, variance components were calculated considering all the factors as random. Variance components were estimated for the combined analysis of AC data from all three environments to get an estimate of G × E. For VS, since there were no repeated measurements, the environments were considered replications for the purpose of variance components estimation. The genomic prediction models were run using BLUEs from each experiment for AC and raw phenotypic data for VS.

Broad-sense heritability (*H*^2^) within environment was estimated as:

(2) $$H^{2}=\frac{\sigma_{G}^{2}}{\sigma_{G}^{2}+\sigma_{e}^{2}\,/\,r}$$

*H*^2^ across environments was estimated as:

(3) $$H^{2}=\frac{\sigma_{G}^{2}}{\sigma_{G}^{2}+\sigma_{G\,E}^{2}\,/\,l+\sigma_{e}^{2}\,/\,l\,r}$$

where $\sigma_{G}^{2}$ is the variance due to genotype, $\sigma_{G\,E}^{2}$ is the variance due to genotype × environment, $\sigma_{e}^{2}$ is the error variance, *l* is the number of environments, and *r* is the number of replications using multi-environment trial analysis.

### DNA Extraction and Genotyping

Genomic DNA was extracted from freeze-dried leaves collected from five individual plants per line using a modified CTAB (cetyltrimethylammonium bromide) method described in CIMMYT laboratory protocols (Dreisigacker et al., 2016) and quantified using a NanoDrop 8000 spectrophotometer V 2.1.0.

The population was genotyped with the 20K Infinium is elect SNP array by TraitGenetics (Gatersleben, Germany). The marker dataset was filtered for polymorphism and minor allele frequency (<5%) and >50% missing data were removed. In addition to these filtering steps, markers with known genetic positions in the genetic map developed by Wang et al. (2014) were extracted for use in GWAS.

### Genome-Wide Association Study

BLUEs from each individual environment were used for GWAS using the mixed linear model (MLM) model implemented in the Genome Association and Prediction Integrated Tool (GAPIT) (Tang et al., 2016). The population structure was assessed via principal component analysis, and the first three components were used as covariates in the population structure (Q) defined by the kinship (K) (Q + K) model. Linkage disequilibrium (LD) decay was assessed by plotting pairwise LD between marker pairs and their genetic distance. Bonferroni correction for multiple testing implemented in GAPIT was used to identify significant associations, which corresponded to a −log_10_(P) value of >5.

### Statistical Models for Genomic Prediction

We used the conventional Genomic Best Linear Unbiased Prediction (GBLUP) extended by the genotype-by-environment interaction term using molecular markers (G × E) and pedigree information (A × E) via the reaction norm model (Jarquín et al., 2014).

#### Baseline Line Model

Consider that the trait performance (*y*_*ij*_) of the *i*th line observed in the *j*th environment can be described as the sum of an overall mean common to all genotypes in all environments μ, plus random deviations as follows:

(4) $$y_{i\,j}=\mu +E_{j}+L_{i}+L\,E_{i\,j}+\varepsilon_{i\,j}$$

where *L*_*i *_ is the random effect of the *i*th line, *E*_*j*_ is the random effect of the *j*th environment, *LE*_*ij*_ is the interaction between the *i*th line and the *j*th environment, and *e*_*ij*_ is the random error term accounting for non-explained variability. The effects are assumed to be independent and identically distributed outcomes following normal densities, such that $E_{i}\,\overset{i\,i\,d}{∼}N\,\left(0,\sigma_{E}^{2}\right)$, $L_{i}\,\overset{i\,i\,d}{∼}N\,\left(0,\sigma_{L}^{2}\right)$, and $e_{i\,j}\,\overset{i\,i\,d}{∼}N\,\left(0,\sigma_{e}^{2}\right)$, while the interaction term from properties of the multivariate density is distributed as follows: $L\,E_{i\,j}\,\overset{i\,i\,d}{∼}N\,\left(0,Z_{E}\,Z_{E}^{\prime }\,\#\,Z_{L}\,Z_{L}^{\prime }\,\sigma_{L\,E}^{2}\right)$ where, $\sigma_{E}^{2}$, $\sigma_{L}^{2}$,$\sigma_{L\,E}^{2}$, and $\sigma_{e}^{2}$ are the associated variance components, and *Z*_*E*_ and *Z*_*L*_ are the incidence matrices that connect phenotypes with environments and lines, and # represents the Hadamar product (cell-by-cell product between two matrices). $Z_{E}^{\prime }$ and $Z_{L}^{\prime }$ are the transpose of the respective incidence matrices.

In the model above, the random effect of the line (*L*_*i*_) can be replaced by *g*_*i*_, which is an approximation of the genetic value of the *i*th line from the genomic relationship matrix. Also the effects of the line (*L*_*i*_) can be replaced by *a*_*i*_, which is the additive effect obtained from the pedigree information. In the models described below, we used either *g*_*i*_ or *a*_*i*_ or both *g*_*i*_ and *a*_*i*_ as well as their interactions with environment *E*_*j*_(*gE*_*ij*,_or*aE*_*ij*_).

#### G × E Models for AC and VS Measured in Environments

We applied a sequence of reaction norm models similar to that used by Jarquín et al. (2014), with genomic-based relationship matrices, and by Pérez-Rodriguez et al. (2015), with pedigree-based relationship matrices. Model 1 included only the main effects of environment (E) and lines (L), whereas model 2 added genomic (G) genomic information to model 1. Model 3 included all three main effects of L, G, and E, and the genomic × environment interactions (G × E). Model 4 included main effects of environment (E), lines (L), and pedigree (A), whereas model 5 added the pedigree × environment (A × E) to the main effect terms of model 4. Model 6 included the main effects of environment (E), lines (L), genomic (G), and pedigree (A). Finally, we fitted model 7, which included all main effects and the two interactions G × E and A × E. A description of the seven models considered in this study is given below.

##### Main Effect Model 1

This simple main effect model considers the response of the *i*th wheat male line and the *j*th environment (*y*_*ij*_) as a function of a random effect model that accounts for only the effect of the environment (*E*_*i*_) and the accession (*L*_*j*_), plus a residual (ε_*ij*_):

(5) $$y_{i\,j}=\mu +E_{i}+L_{j}+\varepsilon_{i\,j}$$

where μ is an intercept, and the random terms remaining are described as in the baseline model. The main effect of environment (*E*_*i*_) models the environment information via the incidence matrix of genotypes (*Z*_*L*_) observed in different environments. In this model, the effects of the lines are regarded as independent; therefore, there is no borrowing of information between untested and tested landrace accessions.

##### Main Effect Model 2

The next main effect model adds in Eq. 5, the random effect of genomic relationship *g*_*i*_, which is an approximation of the true genetic value of the *i*th male wheat line. This approximation is given by the jointly regression on marker covariates $g_{i}=\sum_{m=1}^{p}x_{i\,m}\,b_{m}$, where *x*_*im*_ is the genotype of the *i*th line at the *m*th marker, and *b*_*m*_ is the corresponding effect with the assumption that $b_{m}\,\overset{i\,i\,d}{∼}N\,\left(0,\sigma_{b}^{2}\right)$ (*m* = 1, …, *p*) and $\sigma_{b}^{2}$ is the variance of the marker effects. The vector *g* = (*g*_1_,…,*g*_*I*_)′contains the genomic values of all the lines, and it is assumed to follow a multivariate normal density with zero mean and covariance matrix $C\,o\,v\,\left(g\right)=G\,\sigma_{g}^{2}$, where **G** is the genomic relationship matrix that describes the genomic similarities between pairs of lines and, $\sigma_{g}^{2}$ which is proportional to $\sigma_{b}^{2}$ ($\sigma_{g}^{2}\propto \sigma_{b}^{2}$), the genomic variance. Therefore, main effect model 2 becomes

(6) $$y_{i\,j}=\mu +E_{j}+L_{i}+g_{i}+\varepsilon_{i\,j}$$

where the vector of random effects is assumed $g∼N\,\left(0,G\,\sigma_{g}^{2}\right)$ to be, and the other random effects remain as described The random effects *g* = (*g*_1_,…,*g*_*J*_)′ are correlated such that model 2 allows borrowing of information across *L*_*i*_ tested and untested lines. The genomic matrix **G** given by $G\propto \left(X\,X^{\prime }\right)/\left(2\,\sum_{m=1}^{p}2\,p_{m}\,\left(1-p_{m}\right)\right)$, where *p*_*m*_ is the estimated frequency of the allele whose number of copies at the *i*th accession is counted in *x*_*im*_. Centering (i.e., subtracting 2*p*_*m*_ from the genotype codes) and standardization (i.e., dividing by $\sum_{m=1}^{p}2\,p_{m}\,\left(1-p_{m}\right)$) allows interpreting $\sigma_{g}^{2}=\sigma_{b}^{2}\,\sum_{m=1}^{p}2\,p_{m}\,\left(1-p_{m}\right)$ as a genomic variance. This model does not allow specific genomic effects for each environmental condition but rather a common effect for same lines across environments. Thus, the interaction between markers and environments is introduced in the next model.

##### Main Effect and Interaction Model 3

This model is obtained by extending the main effect model 2 (Eq. 6) to include interaction effects (*gE*_*ij*_) between each marker SNP and each environment. This model can be written as

(7) $$y_{i\,j}=\mu +E_{j}+L_{i}+g_{i}+g\,E_{i\,j}+\varepsilon_{i\,j}$$

where *E*_*j*_, *L*_*i*_, and *g*_*i*_ have already been defined, $\mathrm{gE}∼N\,\left(0,\left(Z_{L}\,G\,Z_{L}^{\prime }\right)\,\#\,\left(Z_{E}\,Z_{E}^{\prime }\right)\,\sigma_{E\,g}^{2}\right)$ is the interaction of the genome with environment, with $\sigma_{g\,E}^{2}$ as the variance component of gE, and the other model terms are as defined previously.

##### Main Effect and Interaction Model 4

Model 4 is similar to model 2 (Eq. 6), but instead of including the random effect of genomic *g*_*i*_, it includes the random effect accounted for by the pedigree *a*_*i*_. This model adds the random effect that incorporates pedigree information by means of the additive relationship matrix (***A***) to model 1 (Eq. 5),

(8) $$y_{i\,j}=\mu +E_{j}+L_{i}+a_{i}+\varepsilon_{i\,j}$$

where *a*_*i*_ is a random additive effect of the line, which in this case accounts for pedigree relationships, where *a* = (*a*,…,*a*_*I*_)′ contains the pedigree values of all the lines and is assumed to follow a multivariate normal density with zero mean and covariance matrix $C\,o\,v\,\left(a\right)=A\,\sigma_{a}^{2}$, where ***A*** is the additive relationship matrix, and $\sigma_{a}^{2}$ is the additive genetic variance. The random effects are correlated such that model 4 allows borrowing between tested and untested lines based on the numerical relationship matrix (***A***). Similarly, this model does not allow specific responses to each environment but instead common effects across environments. Thus, the interaction between lines and environments is introduced in the following model via pedigree information instead of marker data.

##### Main Effect and Interaction Model 5

This model is obtained by extending model 4 (Eq. 8) to include interaction effects (*aE*_*ij*_). Thus, where,*E*_*j*_, *L*_*i*_, and *g*_*i*_ have already been defined, $\mathrm{aE}∼N\,\left(0,\left(Z_{L}\,A\,Z_{L}^{\prime }\right)\,\#\,\left(Z_{E}\,Z_{E}^{\prime }\right)\,\sigma_{a\,E}^{2}\right)$ is the interaction of the genome with environment, with d $\sigma_{a\,E}^{2}$ as the variance component of aE:

(9) $$y_{i\,j}=\mu +E_{j}+L_{i}+a_{i}+a\,E_{i\,j}+\varepsilon_{i\,j}$$

##### Main Effect and Interaction Model 6

Model 6 is similar to model 4 but adds the genomic relationship *g*_*i*_:

(10) $$y_{i\,j}=\mu +E_{j}+L_{i}+a_{i}+g_{i}+\varepsilon_{i\,j}$$

##### Main Effect and Interaction Model 7

This is the complete model with all main effects and interactions:

(11) $$y_{i\,j}=\mu +E_{j}+L_{j}+a_{i}+g_{i}+a\,E_{i\,j}+g\,E_{i\,j}+\varepsilon_{i\,j}$$

where the terms are already defined.

#### Assessing Model Prediction Accuracy by Random Cross-Validation

The described models were fitted in various validation settings to estimate prediction accuracy within an environment (i.e., despite how training and testing set were configured, the correlation between predicted and observed values was computed within environments). For both traits measured, three different validation schemes were studied. We repeated the random cross-validations from Burgueño et al. (2012) and Jarquín et al. (2014) and considered three prediction problems: (1) (CV1) prediction of 20% of wheat lines that have not been evaluated in any environment; (2) (CV2) prediction in incomplete field trials i.e., prediction of performance of lines that have been evaluated in some environments but not in others; and (3) prediction of performance of all lines in an untested environment, using performance data of those lines from correlated environments. CV1 was obtained by assigning accessions to folds; hence, when the phenotype of an accession is predicted, the corresponding training set contains no record of this accession. CV2 was obtained by assigning individual records of each accession to folds; hence, when one is predicting the *i*th line, there are records for the same accession that were part of the training set but observed in a different environment. In both prediction problems, a fivefold cross-validation was performed, where 80% of the accessions formed the training set and 20% of the accessions comprised the testing set for each partition. The assignation of training and testing sets was repeated 20 times (5 × 2 = 100 random partitions) for each one of these cross-validation schemes (CV1 and CV2).

We also evaluated cross-validation CV0, where all lines in one environment were fully predicted by the other environments.

##### Data Repository

The data repository can be found at http://hdl.handle.net/11529/10548495.

## Results

### Phenotypic Variation and Correlation Between Traits

**FIGURE 1:**
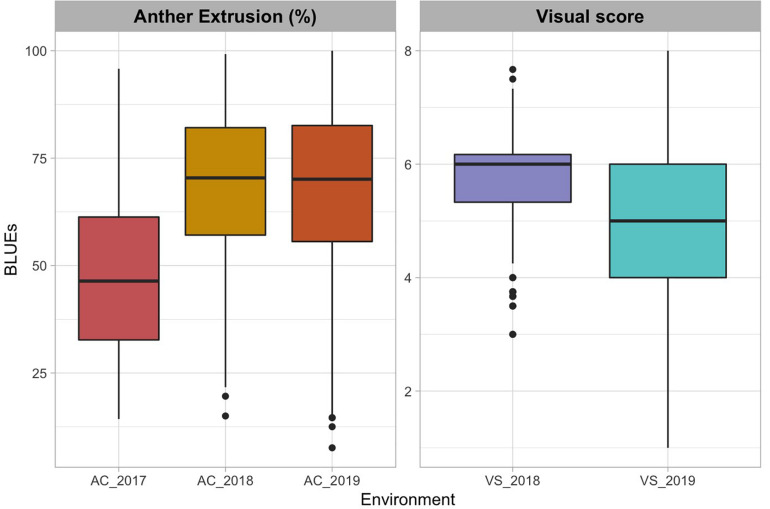
Boxplots of best linear unbiased estimates of anther count (AC) expressed in percentage and visual score (VS) across the three environments (El Batan 2017, Obregon 2018 and El Batan 2019). VS data were collected from only two environments (2018 Obregon and 2019 El Batan). Visual score scale ranges from 0 (0% extruded anthers) to 10 (100% extruded anthers), assessed visually during flowering. Visual score data were not collected in 2017.

The AC in 2017 ranged from 14.3 to 95.8 with an average of 47.2 and a standard deviation (SD) of 18.1 (Figure 1 and Table 1). AC in 2018 ranged from 15.0 to 99.2 with an average of 68.2 and SD of 17.4. The VS in 2018 ranged from 3 to 7 with an average of 5.77 and SD of 0.75. In the 2019 experiment, AC data ranged from 7.61 to 100.00 with an average of 67.58 and SD of 19.44 whereas the VS ranged from 1 to 8 with an average of 4.94 and SD of 1.47.

**TABLE 1:** Variance components and heritabilities for anther count (%) and visual score across three environments (2017, 2018, and 2019).

Trait	Anther count (%)				Visualscore^φ^
		
Env	2017	2018	2019	Combined	2018–2019
Genotype	254.34**	281.92**	330.91**	211.09**	0.29**
Environment	–	–	–	182.70**	–
G × E	–	–	–	80.45**	–
Error	290.82	101.51	191.70	185.03	1.25
Replications	4	5	4	5	2.00
H^2^	0.78	0.93	0.87	0.83	0.32
Grand mean	47.16	68.21	67.57	57.31	5.36

^φ^Visual score data did not have replications within one environment, and data from environments were used as replication for estimating variance components. **p < 0.001.

The genotypic variance was significant for both traits in all three trials (Table 1). The broad-sense heritability estimates for AC ranged from 0.78 to 0.93 across the three environments, whereas the broad-sense heritability for combined VS was 0.32.

The BLUEs of AC were significantly correlated across the three experiments (0.6 –0.67, *p* <0.01) (Supplementary Figure 1). The visual scores across the two environments (2018 and 2019) were also significantly correlated (0.44, *p* <0.01). The VS data were also highly correlated with AC data from all three trials (Supplementary Figure 1).

### Markers Retained After Quality Control

After filtering for polymorphism, minor allele frequency, and missing data, 10,534 markers were retained. The whole marker dataset was used to create a genomic relationship matrix used for GP. For GWAS, the marker dataset was additionally filtered for presence of known genetic positions in the consensus map by Wang et al. (2014). The number of markers retained for GWAS was 7,649.

### Population Structure and Linkage Disequilibrium

**FIGURE 2:**
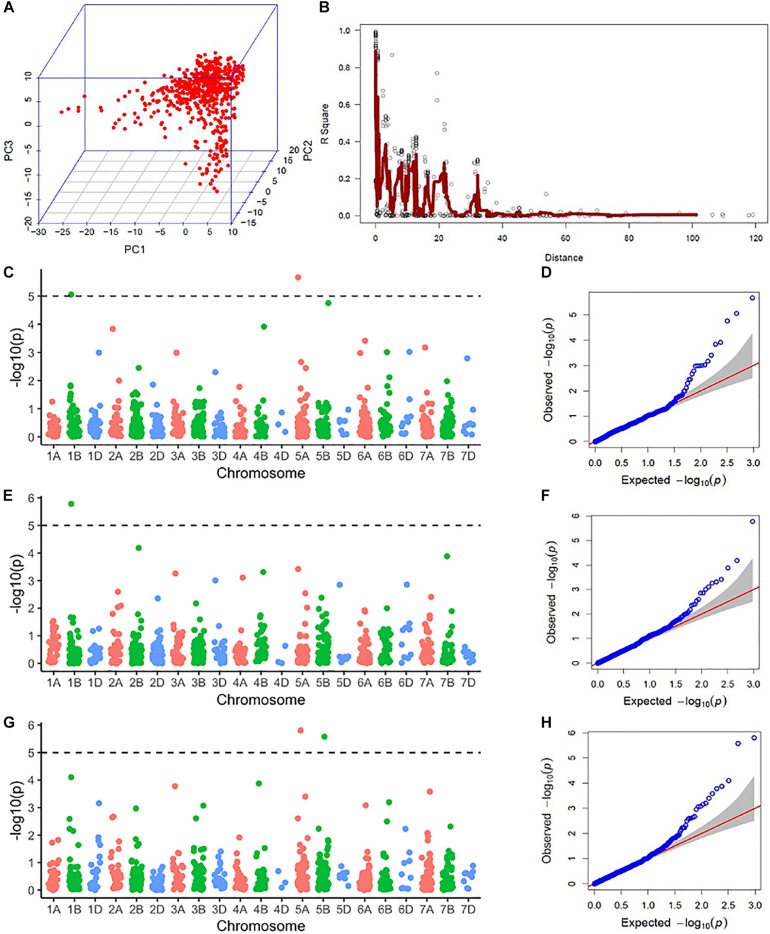
Results from the genome-wide association scan: **(A)** Three-dimensional scatterplot showing the relationship between the first three principal components (PC1, PC2, and PC3) from molecular marker data. **(B)** A pairwise linkage disequilibrium (LD) decay plot with pairwise marker LD in a sliding window of 100 adjacent markers in the y-axis and genetic distance in centimorgans from the genetic map by Wang et al. (2014) in the *x*-axis. The red line indicates a moving average of *r*^2^ values for 10 adjacent markers. **(C)** A Manhattan plot showing −log_10_(P) values of marker trait association (MTA) for anther count (AC) in El Batan (2017) across the genome. The dotted line represents the Bonferroni significance threshold for MTA. **(D)** A quantile-quantile (QQ) plot showing the distribution of expected vs actual −log10(P) values of GWAS using AC from El Batan (2017). **(E)** A Manhattan plot showing −log_10_(P) values of MTA for anther count (AC) in El Batan (2019) across the genome. **(F)** A QQ plot for GWAS using AC data from El Batan (2019). **(G)** A Manhattan plot showing −log_10_(P) values of MTA for visual score (VS) in El Batan (2019) across the genome. **(H)** A QQ plot for GWAS using VS data from El Batan (2019).

Population structure was assessed via principal component analysis. Population structure observed was not very strong since the first three principal components (PCs) only explained 16% of the cumulative variance (Figure 2). Linkage disequilibrium (LD) was assessed by calculating pairwise LD decay over genetic distance (Figure S2). Considering *r*^2^ = 0.2 to be the extent of average intrachromosomal LD, in this population, LD calculated on a sliding window of 100 adjacent markers showed the LD blocks extended up to 25 cM (Figure 2).

### Marker Trait Associations From GWAS

Five marker-trait associations (MTAs) were observed in the GWAS across two environments (2017 and 2019). Of the five MTAs in 2017 and 2019 for VS and AC, two were located in chromosome 1B, two in chromosome 5A, and one in chromosome 5B (Table 2). For AC, three MTAs were observed, two of which were the same and were linked to marker Tdurum_contig75938_1546 (60.62 cM, 62.49 Mb) in 2017 and 2019. This MTA on chromosome 1B had a positive effect on AC, with an allele substitution effect of 5.68–6.84. The two MTAs on chromosome 5A (one each for AC and VS) had a negative effect on AE. SNP marker Ku_c69633_1873 (26.51 cM, 98.94 Mb) decreased the AC by 6.16%, whereas BS00065313_51 decreased the VS by 0.3. The MTA detected on chromosome 5B by the significant SNP marker RAC875_rep_c104919_902 increased the VS by 0.39. For three out of the four markers significantly associated with AC and VS, the favorable allele has a higher frequency in the elite male population we studied (Table 2).

**TABLE 2:** Marker trait associations in the genome-wide association scan (GWAS) for anther count percentage (AC) and visual score (VS) from a field experiment evaluating anther extrusion across three environments (El Batan 2017, Obregon 2018 and El Batan 2019).

				Genetic	Physical					Allele
Env	Trait	SNP	CHR^1^	position (cM)	position^2^ (Mb)	−log_10_(P)	(R^2^)^3^ (%)	Allele^4^	MAF^5^	effects
2017	AC	Ku_c69633_1873	5A	26.51	98.94	5.67	3.2	**A**/G	0.08	–6.16
2017	AC	Tdurum_contig75938_1546	1B	60.62	62.49	5.06	4.6	**A**/G	0.06	5.68
2019	AC	Tdurum_contig75938_1546	1B	60.62	62.49	5.78	3.7	**A**/G	0.06	6.84
2019	VS	BS00065313_51	5A	62.72	492.79	5.81	2.5	**G**/A	0.31	–0.30
2019	VS	RAC875_rep_c104919_902	5B	117.84	594.60	5.58	2.7	A/**C**	0.17	0.39

*A consensus genetic map from Wang et al. (2014) and SNP data from 20K Infinitum SNP assay were used for GWAS. ^1^Chromosome. ^2^Physical position of SNP marker from reference genome of Chinese Spring (Refseq_v1). ^3^Phenotypic variance explained by the SNP in marker trait association. It was calculated by subtracting R^2^ of model without SNP from R^2^ of model with SNP. ^4^Favorable allele is highlighted. ^5^Minor allele frequency (>5%) (Major allele/minor allele).*

### Genome-Wide Prediction

We used seven models of increasing complexity for the genome-wide predictions. The prediction accuracy was assessed using three cross-validation scenarios.

#### Cross-Validation Scenario 1 (CV1)

For CV1, phenotypes of lines that have never been evaluated in the field were predicted using line information, environment information, genomic information, pedigree information, and interaction terms. Prediction accuracies of model 1 (E + L) were negative for both traits under CV1 (Table 3). Upon the addition of genomic information (model 2, E + L + G), the prediction accuracy increased and was in the range of 0.42–0.44 across environments for AC and 0.33–0.46 for VS (Table 3). Inclusion of pedigree information without genomic relationship information decreased the prediction accuracy for both traits. Model 4 (E + L + A) had prediction accuracies in the range of 0.31–0.34 for AC and 0.22–0.34 for VS, which is lower than the prediction accuracies obtained by model 2 for both traits (Table 3). When pedigree information was added in the presence of genomic information, it improved the prediction accuracies. The prediction accuracies for AC were higher for model 6 (E + L + G + A) than those for model 4. In the case of VS, the inclusion of pedigree information along with genomic relationship slightly increased the prediction accuracies in 2018 (Table 3).

**TABLE 3:** Mean and standard deviation of genomic prediction accuracies from the reaction norm models (Jarquín et al., 2014) under the two cross-validation scenarios (CV1 and CV2) for two traits representing anther extrusion; anther count (%) and visual score were collected from a field experiment spanning three environments (El Batan 2017, Obregon 2018 and El Batan 2019).

		Test environments					
		2017		2018		2019	
Traits	Models	Mean	SD	Mean	SD	Mean	SD
		**CV1**					
Anther count (%)	E + L	–0.032	0.036	–0.040	0.034	–0.038	0.028
	E + L + G	0.424	0.014	0.428	0.012	0.441	0.011
	E + L + G + G × E	0.429	0.017	0.435	0.011	0.454	0.012
	E + L + A	0.342	0.013	0.329	0.014	0.302	0.015
	E + L + A + A × E	0.355	0.014	0.333	0.016	0.307	0.016
	E + L + G + A	0.431	0.016	0.427	0.014	0.440	0.014
	E + L + G + A + G × E + A × E	0.441	0.017	0.431	0.015	0.452	0.015
Visual score	E + L			–0.055	0.054	–0.067	0.039
	E + L + G			0.332	0.012	0.459	0.016
	E + L + G + G × E			0.325	0.016	0.461	0.016
	E + L + A			0.217	0.014	0.335	0.017
	E + L + A + A × E			0.216	0.021	0.336	0.018
	E + L + G + A			0.328	0.012	0.458	0.016
	E + L + G + A + G × E + A × E			0.330	0.015	0.448	0.017
		**CV2**					
Anther count (%)	E + L	0.674	0.001	0.704	0.001	0.716	0.002
	E + L + G	0.687	0.001	0.710	0.001	0.726	0.002
	E + L + G + G × E	0.690	0.004	0.722	0.003	0.733	0.004
	E + L + A	0.686	0.001	0.708	0.001	0.715	0.002
	E + L + A + A × E	0.690	0.003	0.718	0.004	0.724	0.003
	E + L + G + A	0.689	0.001	0.708	0.001	0.726	0.002
	E + L + G + A + G × E + A × E	0.694	0.004	0.722	0.004	0.736	0.005
Visual score	E + L			0.424	0.005	0.417	0.007
	E + L + G			0.452	0.007	0.540	0.009
	E + L + G + G × E			0.451	0.011	0.522	0.012
	E + L + A			0.427	0.006	0.496	0.010
	E + L + A + A × E			0.434	0.009	0.454	0.015
	E + L + G + A			0.453	0.006	0.541	0.008
	E + L + G + A + G × E + A × E			0.455	0.011	0.509	0.013

*CV1 is when 20% of lines in the test set do not have any phenotypic record, and CV2 is when the lines in the test set have a phenotypic record from a different environment but not in the environment being tested. The models tested in increasing order of complexity are (E + L): Environment and line main effects; (E + L + G): Environment, line and genomic relationship main effects; (E + L + G + G × E): Environment, line, genomic relationship main effects, and genomic relationship by environment effects; (E + L + A): Environment, line, and pedigree relationship main effects; (E + L + A + A × E): Environment, line, pedigree relationship main effects, and pedigree by environment interaction effects; (E + L + G + A): Environment, line, genomic and pedigree relationship main effects; and (E + L + G + A + G × E + A × E): Environment, line, pedigree relationship, genomic relationship main effects, and genomic relationship by environment and pedigree by environment interaction effects.*

Models with interaction terms generally have slightly higher accuracies as compared to models with main effects only. For example, for both traits model 3 (E + L + G + G × E) had slightly higher prediction accuracy compared to model 2 (E + L + G), and model 5 (E + L + A + A × E) had slightly higher prediction accuracy compared to model 4 (E + L + A). Model 7 (E + L + G + A + G × E + A × E), which is the most complex model, had comparable prediction accuracies for both traits with model 3 (E + L + G + G × E). Compared to model 3, model 7 had higher prediction accuracy only in 2018 for AC and 2019 for VS (Table 3). In most cases, model 3 (E + L + G + G × E) had the highest prediction accuracies among the seven models tested in CV1 for both traits.

#### Cross-Validation Scenario 2 (CV2)

CV2 represents the case of incomplete field trials, where some lines are tested in one environment but are missing in other environments. The phenotypic record of one or more environments is used in conjunction with genomic, pedigree, line, and environment information to predict the missing phenotypic record of lines. Prediction accuracies were higher in CV2 for all seven models across two traits compared to CV1 (Table 3). In CV2, model 1, which had negative prediction accuracy, had comparable prediction accuracies with model 3. Very nominal increments in prediction accuracies were observed with increasing model complexities across both traits. Some variation was observed in prediction across environments. For example, prediction accuracies for AC were higher in 2018 and 2019 compared to 2017. Similarly, prediction accuracies for VS were higher in 2018 compared to 2019. As in CV1, the models predicted AC better than VS in CV2. In addition to higher prediction accuracies, the standard deviation (SD) of prediction accuracies was much smaller in CV2 compared to CV1 (Table 3).

#### Predicting an Untested Environment (CV0)

CV0 is a prediction scenario, where a full dataset from a correlated environment is used to predict the performance of lines in an untested environment. Under CV0, all seven prediction models performed very well across all three environments and two traits (Table 4). The prediction accuracies for AC ranged from 0.68 to 0.69 in 2017, 0.70 to 0.71 in 2018, and 0.71 to 0.73 in 2019 (Table 4). The prediction accuracies for VS ranged from 0.43 to 0.47 in 2018 and 0.43 to 0.48 in 2019 (Table 4). The differences that were apparent between model 2 and model 4 via the inclusion of pedigree vs. genomic relationship in CV1 were not apparent in CV0. Similarly, the inclusion of interaction effects also did not make as much difference as it did in CV1.

**TABLE 4:** Mean and standard deviation of genomic prediction accuracies from the reaction norm models (Jarqin et al., 2014) under the cross-validation scenarios (CV0) for two traits representing anther extrusion; anther count (%) and visual score were collected from a field experiment spanning three environments (2017, 2018, and 2019).

		Test environments		
		2017	2018	2019
Traits	Models	Mean	Mean	Mean
		**CV0**		
Anther extrusion (%)	E + L	0.676	0.706	0.718
	E + L + G	0.686	0.703	0.722
	E + L + G + GE	0.683	0.705	0.724
	E + L + A	0.684	0.705	0.710
	E + L + A + AE	0.677	0.707	0.716
	E + L + G + A	0.687	0.701	0.720
	E + L + G + A + GE + AE	0.681	0.701	0.725
Visual score	E + L		0.432	0.433
	E + L + G		0.465	0.470
	E + L + G + GE		0.453	0.450
	E + L + A		0.434	0.464
	E + L + A + AE		0.434	0.435
	E + L + G + A		0.463	0.482
	E + L + G + A + GE + AE		0.462	0.470

*CV0 is prediction of performance of lines in a new environment using a phenotypic record from other environments. The models tested in increasing order of complexity are: (E + L): Environment and line main effects; (E + L + G): Environment, line, and genomic relationship main effects; (E + L + G + G × E): Environment, line, genomic relationship main effects, and genomic relationship by environment effects; (E + L + A): Environment, line, and pedigree relationship main effects; (E + L + A + A × E): Environment, line, pedigree relationship main effects, and pedigree by environment interaction effects; (E + L + G + A): Environment, line, genomic, and pedigree relationship main effects; and (E + L + G + A + G × E + A × E): Environment, line, pedigree relationship, genomic relationship main effects, and genomic relationship by environment and pedigree by environment interaction effects.*

## Discussion

The success of hybrid wheat breeding depends on reduced costs for hybrid seed production and grain yield heterosis. The presence of heterosis of grain yield in hybrid wheat has already been established over several decades, while newer studies have suggested, in addition, that the development of heterotic pools could increase the level of heterosis (Longin et al., 2013; Zhao et al., 2015; Rembe et al., 2019). Reducing the cost of hybrid seed production appears to be a more complex challenge. Methods of hybrid seed production, such as cytoplasmic male sterility, genetic male sterility, and chemical hybridization methods, need to be optimized. In addition, the floral biology of wheat needs to be redesigned to favor cross-pollination (Whitford et al., 2013; Boeven et al., 2016). The most important factor to facilitate cross-pollination in wheat is higher AE, while ensuring that the male parent does not lose its ability to contribute to higher grain yield in the subsequent hybrid crosses. A recurrent selection scheme needs to be implemented within the male germplasm pool to develop superior male lines with desirable floral traits favoring cross-pollination along with other attributes for superior yield and quality. In the absence of large-effect loci, high G × E variance and labor-intensive phenotyping, MAS, and visual selection are inadequate. Hence, in this study, we demonstrate the utility of genome-wide prediction for AE via modeling for G × E and environmental covariates as a more reliable substitute for MAS and/or visual selection.

### Phenotypic Evaluation of AE

AC and VS appear to be reliable measurements for AE, as demonstrated by their high heritability estimates. The heritability of AC in this study ranged from 0.79 to 0.93 which is comparable to heritability reported in similar previous studies (Boeven et al., 2016; Muqaddasi et al., 2017a). The heritability of VS in this study was quite modest compared to similar previous studies. Previous studies have reported heritabilities in the range of 0.5 to 0.8 for VS (Boeven et al., 2016; Muqaddasi et al., 2019). However, it should be noted that the error variance for VS was confounded with G × E due to the lack of replications within an environment. High positive correlations between VS and AC within the same environment indicated that VS could be a reliable trait for measuring AE. VS has been found to be an adequate trait to measure AE in several previous studies (Boeven et al., 2016, 2018; Muqaddasi et al., 2017c,a). The continuous distribution of AC indicated that AE is a complex trait governed by cumulative effects of numerous minor effect loci, making it suitable for GS. Moreover, significant genotypic variances for the two traits measured indicated that these traits can be improved by breeding efforts (Table 1; Boeven et al., 2016, 2018).

### Marker Trait Associations for AE

Height-reducing loci, such as *Rht-B1* and *Rht-D1*, have been shown to reduce AE in several previous studies (Boeven et al., 2016; Würschum et al., 2018). None of the large effect *Rht* loci were identified in our analysis. CIMMYT spring wheat germplasm has been subjected previously to GWAS for AE and, similarly, *Rht* loci were not identified (Muqaddasi et al., 2017c). This is most likely due to the fact that *Rht* loci, in particular *Rht-B1*, have been largely deployed in CIMMYT since the 1970s, and Rht-B1 is almost fixed in recent elite germplasm (Aisawi et al., 2015; Basnet et al., 2019).

Two MTAs were identified in chromosome 5A, one on the distal and one on the proximal end of the chromosome, based on physical positions. Previous studies have reported QTL for AE with minor to moderate effects on chromosome 5A via linkage mapping in biparental populations (Lu et al., 2013; Buerstmayr and Buerstmayr, 2015; Muqaddasi et al., 2019). The previously reported QTL for AE are spread throughout chromosome 5A, and the MTAs in 5A share close proximity with several of these previously reported QTLs. For example, the MTAs identified in this study in the short arm of 5A is at 26.51 cM (98.94 Mb), which is in between the AE QTL identified by Buerstmayr and Buerstmayr (2015) at 20 cM and Lu et al. (2013) at 33 cM. Similarly, Muqaddasi et al. (2019) have reported AE QTLs in CIMMYT germplasm at 59 cM, which lies very close to the other MTA identified in this study at 62.72 cM (492.79 Mb). Several previous studies have also reported QTL for AE on 1B and 5B (Skinnes et al., 2010; Boeven et al., 2016; Muqaddasi et al., 2017b,a). Boeven et al. (2016) have reported an MTA for AE in 1B at 74.4 cM; Muqaddasi et al. (2017b) have reported an MTA at 56.4 cM; Muqaddasi et al. (2017a) have reported an MTA at 70.08 cM; and Skinnes et al. (2010) have reported a QTL with confidence interval of 86–102 cM. The MTA detected in 1B in this study at 60.62 cM lies close enough to these previously reported loci. Similarly, in 5B Muqaddasi et al. (2017b) have reported an MTA at 108.7 cM, whereas we identified an MTA at 117.84 cM in this study.

Since the phenotypic effects of these MTAs across studies were low, there is limited scope to use these MTAs in MAS. The only option might be to use these QTL together with the *Rht* loci in MAS, in the event that they can be successfully validated and concurrently do not show any negative effect on other traits, e.g., lodging or grain yield.

Despite having high heritabilities for VS and AC, the MTAs explain a very low amount of phenotypic variance. This signifies the highly polygenic nature of inheritance of AE and suggests that MAS is not the best strategy for driving genetic gains in AE. In this context, genomic prediction/selection can be an excellent strategy for making selection gains in breeding for AE.

### Genome-Wide Predictions

Genome-wide prediction for AE has been previously conducted on an unrelated smaller subset of CIMMYT spring wheat germplasm (Muqaddasi et al., 2017c) and winter wheat germplasm from Western Europe (Boeven et al., 2016). Both studies used single-trait models without explicitly modeling G × E. Incorporating G × E effects and environmental covariates has previously shown higher prediction accuracy for grain yield (Burgueño et al., 2012; Jarquín et al., 2014; Sukumaran et al., 2017, 2018; Krause et al., 2019), micronutrient concentration (Velu et al., 2016), and lint yield in cotton (Pérez-Rodríguez et al., 2015). In this study, we attempted to implement the same approach for predicting AE.

#### Prediction of Performance of Untested Lines

In CV1, lines that were never tested before were predicted by borrowing information from closely related individuals, which is akin to the previous GS studies for AE (Boeven et al., 2016; Muqaddasi et al., 2017c). Boeven et al. (2016) reported prediction accuracies of 0.3 for VS and 0.6 for AC implementing ridge regression BLUP (RR-BLUP), whereas inclusion of weighted effects of *Rht* loci via weighted ridge regression BLUP (wRR-BLUP) increased the prediction accuracies to 0.5 for VS and 0.7 for AC. Muqaddasi et al. (2017c) reported a prediction accuracy of 0.6 for VS in a CIMMYT population using RR-BLUP. However, the prediction accuracy was standardized with the square root of heritability in the Muqaddasi et al. (2017c) study. The prediction accuracies of the main effect model (model 2) in CV1 for both VS and AC are comparable to these previous studies. When interaction effects were added in models 3 and 7, the prediction accuracies tended to increase slightly (1–3%). In CV1, models using the G matrix (models 2 and 3) always had higher prediction accuracies than models using pedigree-based A matrix (models 4 and 5), which has been reported previously by Campos et al. (2009, 2010) and Crossa et al. (2010, 2011).

In model 1, where only the main effect of environments and lines are used for prediction, negative prediction accuracy is observed. Since neither pedigree information nor genomic information is included in this model, it does a very poor job of predicting performance. This is expected since genomic prediction is based on using information borrowed from related individuals via pedigree and genomic relationship, and in model 1 only incidence matrices for lines and environments are included. Once we start including pedigree and genomic information the prediction accuracies are positive and higher.

#### Prediction of Performance of Previously Tested Lines in Untested Environments

CV2 and CV0 were the scenarios where phenotypic information of the same line from one environment was used to predict VS and AC in another environment. CV2 is similar to what is also called sparse testing, where some of the lines are missing, whereas CV0 is the prediction of the whole population in a previously untested environment. In cases when a phenotypic record of the line being tested is used to train the model (CV2 and CV0), the prediction accuracy is higher compared to the case where the line has never been tested previously (CV1) (Sukumaran et al., 2017, 2018; Basnet et al., 2019). We found similar results in the study, as expected. These CV scenarios can be very useful in the case of traits that are difficult to phenotype due to cost or the labor-intensive nature of phenotyping. The results for CV0 and CV2 indicate that untested sites, environments, and years can be predicted with high reliability. CV0 and CV2 scenarios can supplement the field evaluation efforts of breeding programs. In particular, sparse testing, i.e., the CV2 scenario, is already in practice in hybrid breeding for the development of heterotic pools in wheat (Zhao et al., 2015).

Inclusion of interaction effects such as G × E and A × E produced mixed results. For AC, the inclusion of interaction effects produced a very nominal increase in prediction accuracy (1–3%), whereas for VS it decreased nominally in most cases.

### Implications for Hybrid Wheat Breeding

GS is promising for driving the genetic gain of AE. However, the prediction accuracies are also dependent on trait heritability values (Velu et al., 2016; Acosta-Pech et al., 2017). AC had higher heritability compared to VS in our study. VS is easier to phenotype than AC. Here, VS data were unreplicated, and the error variance was confounded with G × E. It is easier and cost-effective to increase replications for VS than to collect data for AC routinely in the breeding program. Hence, the use of replicated trials can help increase the prediction accuracy for VS.

Inclusion of interaction terms had a very nominal advantage in prediction accuracy for AC, whereas it was sometimes counterproductive in VS, most likely due to increasing model complexity. Based on observations from this study, it is possible to predict AE with reasonable accuracy using pedigree data only, but the inclusion of genomic data should always be preferred. Inclusion of both pedigree and genomic data appears to work best but not in all cases.

Reciprocal recurrent selection is a promising strategy in hybrid wheat breeding (Rembe et al., 2019). For VS, sparse testing appears to be a good strategy, where a subset of lines would be tested in some environments but not all. Information on tested relatives in an environment can be used to predict untested lines. For AC, which is labor-intensive and expensive to phenotype, data from a subset of highly maintained trials can be used to predict performance in an untested environment.

## Data Availability Statement

The datasets for this study can be found in the CIMMYT Research Data & Software Repository Network (http://hdl.handle.net/11529/10548495).

## Author Contributions

AA collected and analyzed the data, and wrote the manuscript. BB conceived and designed the experiment, collected and analyzed phenotypic data, and wrote the manuscript. JC and DJ analyzed the data and wrote the manuscript. PB and FC did field experiments, collected phenotypic data, and reviewed the manuscript. SD generated and curated genotypic data, analyzed data, and reviewed the manuscript. YM and AI developed the concept and reviewed the manuscript. All authors contributed to the article and approved the submitted version.

## Conflict of Interest

YM was employed by company Syngenta France S.A.S, France. The remaining authors declare that the research was conducted in the absence of any commercial or financial relationships that could be construed as a potential conflict of interest.

## Supplementary Material

Supplementary Figure 1 Plot showing pairwise correlations between traits measuring anther extrusion from three environments (El Batan 2017, Obregon 2018 and El Batan 2019). Trait abbreviations: AE_2017: Anther count (%) 2017; AE_2018: Anther count (%) 2018; AE_2019: Anther count (%) 2019; VS_2018: Visual score of anther extrusion 2018; and VS_2019: Visual score of anther extrusion 2019. Visual score data were not collected in 2017.
