# Supplementary figures and images for: Genome-Wide Association Mapping and Genomic Prediction of Anther Extrusion in CIMMYT Hybrid Wheat Breeding Program via Modeling Pedigree, Genomic Relationship, and Interaction With the Environment
Source: Front Genet. 2020 Dec 8;11:586687. doi: 10.3389/fgene.2020.586687 (PMC7755068; doi:10.3389/fgene.2020.586687)

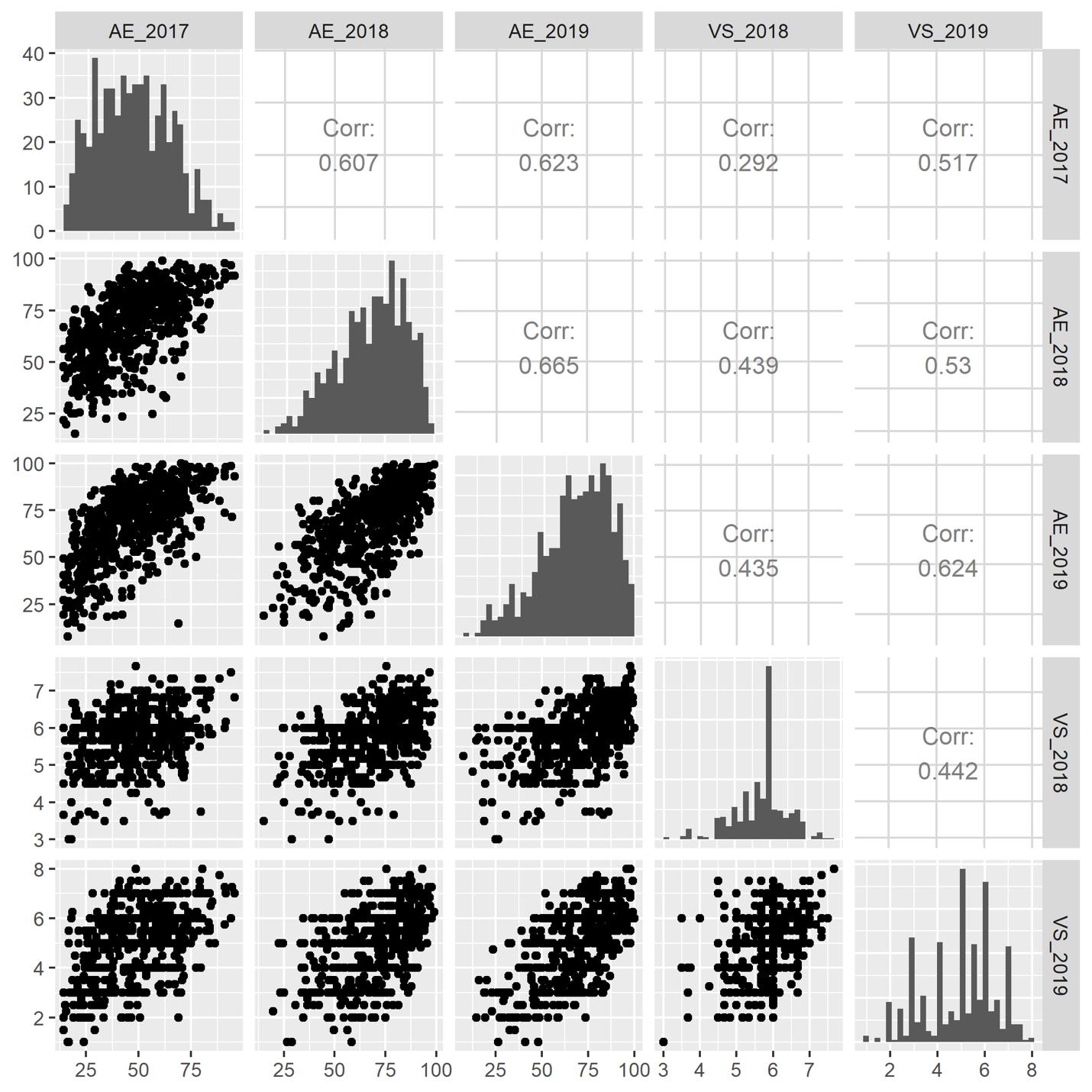

Supplement: Supplementary Figure 1 — Plot showing pairwise correlations between traits measuring anther extrusion from three environments (El Batan 2017, Obregon 2018 and El Batan 2019). Trait abbreviations: AE_2017: Anther count (%) 2017; AE_2018: Anther count (%) 2018; AE_2019: Anther count (%) 2019; VS_2018: Visual score of anther extrusion 2018; and VS_2019: Visual score of anther extrusion 2019. Visual score data were not collected in 2017. [file Image_1.TIF]
